# Additive Manufacturing of Advanced Ceramics Using Preceramic Polymers

**DOI:** 10.3390/ma16134636

**Published:** 2023-06-27

**Authors:** Jinchen Han, Chang Liu, Robyn L. Bradford-Vialva, Donald A. Klosterman, Li Cao

**Affiliations:** 1 Department of Chemical and Materials Engineering, University of Dayton, Dayton, OH 45469, USA; hanj07@udayton.edu (J.H.); dklosterman1@udayton.edu (D.A.K.); 2 Technical Center, Nippon Paint Automotive Americas, Inc., Cleveland, OH 44102, USA; xiandaihanyucidian@gmail.com; 3 Air Force Research Laboratory (AFRL/RXMD), Manufacturing & Industrial Technologies Division, Wright-Patterson AFB, Dayton, OH 45433, USA; robyn.vialva.1@us.af.mil

**Keywords:** ceramic material, preceramic polymer, feedstock material, additive manufacturing, 3D printing

## Abstract

Ceramic materials are used in various industrial applications, as they possess exceptional physical, chemical, thermal, mechanical, electrical, magnetic, and optical properties. Ceramic structural components, especially those with highly complex structures and shapes, are difficult to fabricate with conventional methods, such as sintering and hot isostatic pressing (HIP). The use of preceramic polymers has many advantages, such as excellent processibility, easy shape change, and tailorable composition for fabricating high-performance ceramic components. Additive manufacturing (AM) is an evolving manufacturing technique that can be used to construct complex and intricate structural components. Integrating polymer-derived ceramics and AM techniques has drawn significant attention, as it overcomes the limitations and challenges of conventional fabrication approaches. This review discusses the current research that used AM technologies to fabricate ceramic articles from preceramic feedstock materials, and it demonstrates that AM processes are effective and versatile approaches for fabricating ceramic components. The future of producing ceramics using preceramic feedstock materials for AM processes is also discussed at the end.

## 1. Introduction

Ceramic materials are a diverse group of nonmetallic compounds with a long and fascinating history dating back to 25,000 BCE [1]. Since then, they have played essential roles in the evolution and development of human civilization [2]. Because of their strong atomic bonding nature (ionic or covalent bonding), ceramic materials possess exceptional physical, chemical, thermal, mechanical, electrical, magnetic, and optical properties and exhibit a wide range of applications in various industries, including aerospace, automotive, defense, infrastructure, energy, healthcare, consumer goods, and sensor [3]. Commonly, ceramic materials can be divided into two main categories: traditional and advanced (or technical). Traditional ceramics are those made from naturally occurring materials, such as clay and sand [1,4]. In contrast, advanced ceramics are typically synthesized using advanced manufacturing techniques, and examples include silicon carbide (SiC), silicon nitride (Si_3_N_4_), boron nitride (BN), aluminum oxide (Al_2_O_3_), zirconium oxide (ZrO_2_), composites, and many others [4]. With a precisely controllable production process for manipulating the composition and, thus, the microstructure, advanced ceramic materials can have exceptional properties highly desirable for widespread applications far beyond those of traditional ceramic materials [5]. Therefore, advanced ceramic materials are among the most important materials demanded in modern industries. For example, the core components of insulators for hypersonic aircraft, tissue engineering, and micro- and nanoelectromechanical systems heavily rely on advanced ceramics [6,7,8,9,10]. Recently, some reports indicated that the global advanced ceramic market is expected to grow at a compound annual growth rate (CAGR) of 5.4% from 2022 to 2032 [11].

The increased need for advanced ceramic materials and their functional products requires advanced manufacturing technologies to meet specific applications. However, high-quality ceramic materials are often challenging to fabricate because of their inherent physical and chemical properties. Unlike metal or polymer, ceramic materials usually have low toughness and ductility because of their atomic bonding nature, making them more prone to cracking and fracturing when subjected to mechanical or thermal stress or impact during manufacturing [12]. In particular, small defects or cracks inside ceramics can propagate and cause sudden failure of the product [13]. Many attempts have been conducted to improve the quality and properties of ceramic products, especially the development of ceramic matrix composites (CMCs) [14].

**Figure 1:**
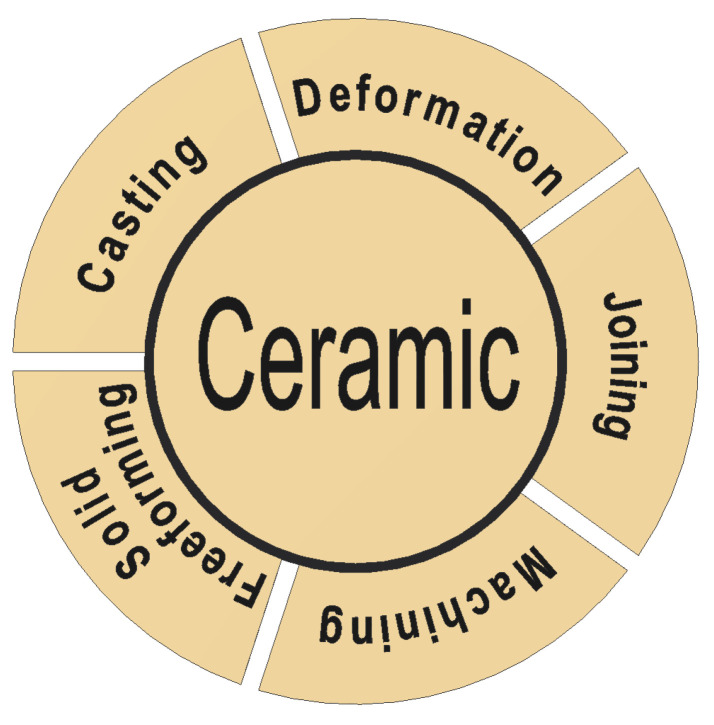
Illustration of the manufacturing technologies for ceramics.

In addition to exploring and developing advanced ceramic materials, there is a growing need to fabricate these materials into intricate geometric structures that meet strict performance criteria and functional requirements [15]. Ceramic materials usually have a high hardness and high melting temperature, increasing the challenges to machine or shape their final geometry and dimensions. Therefore, the successful fabrication of high-quality advanced ceramic materials and their products necessitates the adoption of appropriate advanced manufacturing technologies for various applications. Numerous advanced manufacturing technologies for ceramics have been reported in the literature [16,17,18]. Evans proposed a classification scheme that groups these technologies into five distinct categories: (i) casting/solidification processing; (ii) deformation processing; (iii) machining/material removal; (iv) joining; and (v) solid freeforming, as shown in Figure 1 [19,20]. Most conventional fabrication methods for ceramic materials fall into the first four categories. Solid freeforming encompasses a range of advanced fabrication processes in which the solid structure and shape are created by depositing materials point by point, line by line, or layer by layer [19].

**Figure 2:**
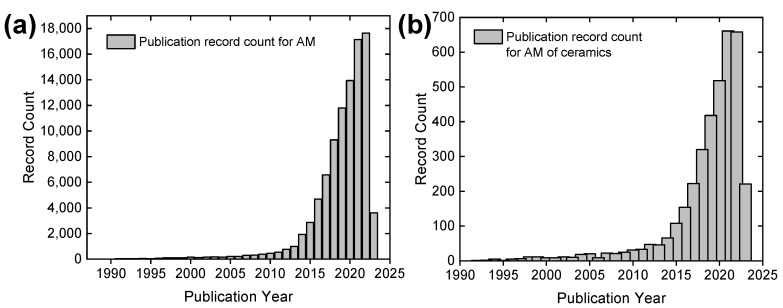
Annual publication records identified in the Web of Science database: (**a**) search results using the topics “additive manufacturing” or “3D printing” between 1987 (first record) and April 2023; (**b**) search results using the refined keyword “ceramic” in (**a**).

Additive manufacturing (AM), also known as 3D printing or rapid prototyping, is one kind of solid freeforming process that involves adding material to create physical objects layer by layer. AM is an innovative manufacturing technique and has become an integral part of Industry 4.0, representing the Fourth Industrial Revolution characterized by the integration of automation, analysis, data exchange, and process through innovative technologies in manufacturing processes [21]. AM was first developed in the 1980s [22]. Since then, it has undergone fast growth, particularly over the last decade. This surge in growth can be attributed to the rapid development of advanced technologies [23,24]. Figure 2a presents the scientific publication record related to AM over the last three decades. The data were accessed from Web of Science with the searched keywords “additive manufacturing” and “3D printing” in the selected “Topic” options. AM has numerous advantages compared to other manufacturing technologies, such as the flexibility to modify designs, accommodate complex geometries, save materials, shorten production time for prototypes, and reduce costs [25,26,27]. As a result, AM has been adopted for product fabrication using different types of materials, including polymer, metals, ceramics, and composites for applications in various industries, such as aerospace, automotive, defense, infrastructure, healthcare, consumer goods, toys, art, and food [28,29]. It is reported that the global 3D printing market size is projected to grow at a CAGR of over 20% for the next decade [30].

As mentioned previously, the fabrication of ceramic products can be challenging because of their low toughness and ductility, high hardness, and susceptibility to defects or cracks. AM has shown great potential in addressing these limitations and unlocking new possibilities in the design and fabrication of ceramic components. Following the development trend of AM-related research, AM for ceramic materials has also experienced similar rapid development, as shown in Figure 2b [31,32,33]. Multiple AM methods can be used to produce ceramic materials, which can be classified based on the type of feedstock materials or the AM techniques adopted. The AM techniques for ceramic materials are discussed in later sections. Based on the type of feedstock materials, ceramic powder with polymer (i.e., binder) and preceramic polymers (with or without fillers) are the two most popular materials for AM processes [34,35,36,37,38,39,40]. In detail, AM processes with ceramic powder and polymer involve mixing ceramic powders with binder polymers for the 3D fabrication of solid objects. Once printed, objects usually undergo a sintering process to remove the polymer and fuse the ceramic powder and, finally, the solid ceramic objects are produced [41]. The AM processes with preceramic polymers involve feedstock materials that usually consist of organic compounds and possess excellent processability, which allows them to be easily shaped and molded into desired geometric structures. After pyrolysis, the AM-fabricated preceramic physical objects will be further converted into functional ceramic components [42]. This kind of AM utilizing preceramic polymers for fabricating ceramic materials can be grouped into one of the polymer-derived ceramics (PDCs) processes.

AM processes have been adopted for fabricating advanced ceramic materials, such as CMCs, particularly AM processes using preceramic polymers, because of their excellent processibility for forming complex structures and shapes, as mentioned earlier [41] The CMCs are composites with reinforcement material embedded in a ceramic matrix. CMCs can overcome the previously discussed ceramic limitations and offer improved properties because of the synergistic combination of properties from the reinforcement and matrix materials [43,44]. Therefore, CMCs have many critical applications and are highly demanded in various fields, including aerospace, defense, energy and power, electrical and electronics, and more [43]. A recent report indicated that the global ceramic matrix composites market size was expected to grow at a CAGR of 12.8% from 2023 to 2030 [45]. Typically, reinforcement materials (also known as fillers) in CMCs have one or more superior properties than the matrix. Combining a ceramic matrix with fillers achieves enhanced properties that exceed those of the individual constituents alone [46]. CMCs can be classified according to the reinforcement material’s type and dimension/shape. Composites, including reinforcement materials at the micro size or larger, are usually called CMCs. For example, carbon fibers, SiC fibers, or microscale particles have been extensively used to fabricate CMCs for aerospace applications [39,40]. Composites using zero-, one-, or two-dimensional nanomaterials as reinforcements are well known as ceramic matrix nanocomposites (CMNCs) [47]. For example, nanoparticles (such as Y_2_O_3_, Al_2_O_3_, SiC, Si_3_N_4_, and nanodiamonds); nanotubes and nanofibers (such as carbon nanotubes, nanofibers, and SiC nanofibers); and nanosheets (such as graphene, boron nitride, and WS_2_) have been used as reinforcement materials to fabricate CMNCs with improved mechanical, thermal, or electrical properties [48,49,50,51,52,53,54].

AM processes using preceramic feedstock materials have been demonstrated to be effective and versatile approaches for manufacturing functional advanced ceramic materials with complex geometric structures. They have been investigated for various applications, especially in the last decade [3,55]. However, the use of preceramic polymers as a start for 3D printing is still developing. Therefore, it is necessary to review the current state of various AM process technologies for fabricating ceramic components using preceramic feedstock materials.

## 2. Preceramic Feedstock Materials for Additive Manufacturing

**Figure 3:**
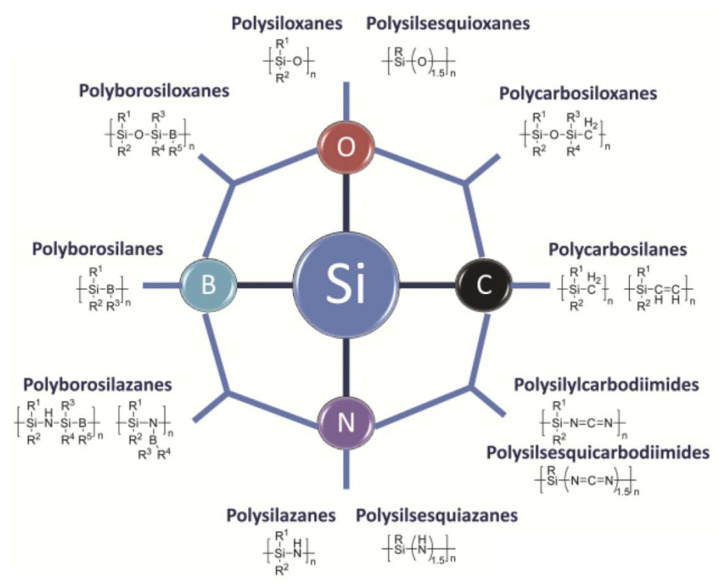
Main classes of Si-polymer precursors for ceramics. Adapted from Reference [59] with permission. Copyright 2010 John Wiley and Sons.

Preceramics are a type of material that serve as ceramic precursors and can be converted to ceramics after pyrolysis in an inert or reactive atmosphere [56]. Ceramic materials formed in this way are known as polymer-derived ceramics, or PDCs, first discovered in the 1960s [57]. PDCs can exhibit a wide range of compositions depending on the makeup of preceramic polymers, such as silicon-, aluminum-, and boron-containing polymers [58]. Well-known preceramic polymers contain a primary Si backbone and usually consist of C, O, N, B, and H atoms, such as polysiloxanes, polysilazanes, and polycarbosilanes [59]. Figure 3 shows the common Si-based polymers. These kinds of silicon-based preceramic polymers can be converted to various types of ceramics, such as silicon carbide (SiC), silicon oxide (SiO_2_), silicon oxycarbide (SiOC), and silicon carbonitride (SiCN), after pyrolysis [60,61,62,63]. Compared to ceramic powders, preceramic polymers offer much more flexibility for effectively fabricating ceramic components with complex geometric structures and shapes using a wide range of processes, such as casting, molding, and AM [3,64]. Preceramic polymers can have different configurations/microstructures, which can affect the composition, microstructure, porosity, yield, and properties of fabricated ceramics [65]. Common silicon-based preceramic polymers, such as polycarbosilanes, polysiloxanes, polysilazanes, and polyborosilazanes, have already been adopted for AM processes to produce ceramic components and will be discussed in the following sections.

## 3. Additive Manufacturing for Preceramic Polymers

### 3.1. Additive Manufacturing Processes

**Figure 4:**
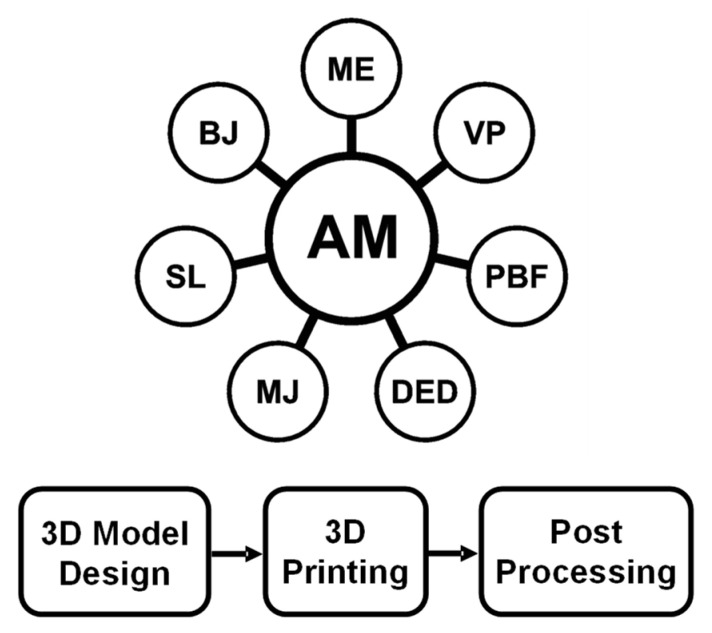
Seven categories of AM processes (**upper**), and the three primary steps of a typical AM process (**lower**).

Currently, numerous kinds of 3D printing systems are available on the market. According to the American Society for Testing and Materials (ASTM), AM processes can be classified into seven categories: vat photopolymerization (VP), material extrusion (ME), powder bed fusion (PBF), material jetting (MJ), binder jetting (BJ), directed energy deposition (DED), and sheet lamination (SL), as shown in Figure 4 (upper) [66]. All seven AM methods are layer-by-layer printing processes to fabricate solid structural components. The main differences between these techniques are feedstock material form and how they are used to form layers. Briefly, VP uses liquid photopolymers that are selectively cured by light. With ME, materials are extruded from a nozzle or orifice and form a solid structure, and as the name implies, PBF uses a material powder bed where powder is selectively fused together. In MJ, liquid material is selectively jetted from a print head to build parts. Different from MJ, BJ utilizes a liquid bonding agent to selectively deposited to bind powders together. With DED, powder or filaments are thermally fused together when they are being deposited. In SL, material sheets are bonded together to form layered structure [66].

Even though there are seven AM process categories, their principles are similar. Typically, any AM process involves three primary steps (Figure 4 (lower)): (i) a digital model design—a process in which computer-aided design (CAD) software is utilized to create a 3D digital model; (ii) 3D printing—a manufacturing process in which the digital model is transferred to a 3D printer, and the physical object is printed using various parameters; and (iii) post processing—a process that encompasses activities, such as support removal, cleaning, surface finishing, heat treatment, or other necessary steps to ensure that the final product meets the desired specifications.

### 3.2. Additive Manufacturing Processes for Preceramic Polymers

**Figure 5:**
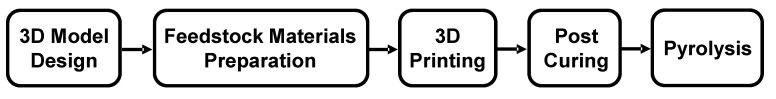
A typical AM process for fabricating ceramic components using preceramic feedstock materials.

As discussed earlier, any AM process involves three primary steps (Figure 4). Different AM processes may also require additional steps to complete the fabrication process. For example, powder mixing before printing is involved in fabricating carbon nanotube-reinforced metal matrix composites using a laser PBF process [67]. Similarly, the AM process for preceramic polymers involves other steps before the final ceramic products are printed. Since the use of preceramic materials as feedstocks for fabricating ceramic products is still developing, commercially available preceramic polymers specifically designed for AM are rarely available on the market. Therefore, other steps, including feedstock material preparation, are needed. Preparing the material involves mixing the preceramic polymer with additives, such as coupling agents like photo- or thermal initiators or reinforcement materials, to create a printable mixture of starting material. Once prepared, the material is loaded into a 3D printer to build solid parts that are called green parts. Here the “green” means the solidified preceramic component after printing and before pyrolysis. After printing, the green objects are post-cured to remove any remaining uncured resin and ensure its structural stability and integrity. This is usually conducted by exposing the object to light or heat, depending on the coupling agents used. The next step is pyrolysis, where the green objects are heated to a specific temperature under an inert or reactive atmosphere to convert them into ceramic objects. Finally, the pyrolyzed objects may be subjected to post-processing (e.g., polishing and coating) or other treatments, such as chemical vapor infiltration (CVI) and polymer infiltration and pyrolysis (PIP) to improve the appearance and performance [68,69]. A typical AM process for manufacturing ceramic components using preceramics is illustrated in Figure 5. Theoretically, all seven AM methods can be used to produce ceramic components. However, only a few of them have been adopted for use with preceramic polymers, and they are discussed in the following sections.

#### 3.2.1. Vat Photopolymerization

**Figure 6:**
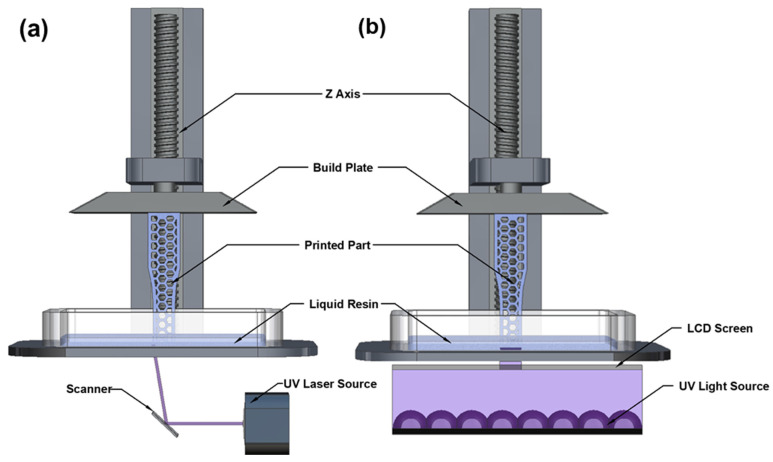
Schematic diagram of SLA and DLP 3D printers: (**a**) SLA; (**b**) DLP.

As mentioned earlier, VP is an AM process in which light-sensitive liquid photopolymers in a vat are selectively cured with a light source layer by layer to create a desired solid structure. VP can be a bottom-up or top-down 3D-printing approach depending on part position on the build plate and the light source [70]. The most common VP are stereolithography (SLA) and digital light processing (DLP). Their main difference is how the photo resin is cured with a light beam. With SLA, a focused light beam (usually a UV laser) is used. The laser is directed by a set of mirrors to be precisely focused on the resin, curing it layer by layer to create the final solid object. SLA 3D printing is particularly beneficial for creating high-precision parts with intricate details. Figure 6a shows a schematic diagram of the SLA printing process. With DLP, the light beam is expanded like a projector to cure one layer at a time, forming a layer much faster than SLA and decreasing the print time. In the current DLP systems, a liquid crystal display (LCD) projector is widely used as the light source to achieve high-resolution illuminations on the photoresin [71]. Figure 6b shows an example of a bottom-up DLP process using an LCD projector. DLP is a more economical 3D printing technique compared to SLA and, therefore, has been adopted for most VP processes.

Other advanced VP processes include continuous liquid interface production (CLIP) and two-photon polymerization (2PP) [72,73,74,75]. CLIP is a proprietary process where a light source selectively cures a photosensitive liquid resin that is continuously flowing during printing. Unlike other VP processes, a UV transparent dead zone is created by an oxygen-permeable window (oxygen-impaired photopolymerization region) between the light projector and the resin layer that needs to be cured [74,76]. The continuous printing nature of CLIP is key to printing layerless parts compared to other VP processes [77]. However, the lateral resolution of CLIP is usually constrained because of the limited pixel size of the LCD light [76]. Recent research has shown improved lateral resolution through a combination of the CLIP technology with a reduction lens optics system and an in-line camera system [78]. Hsiao et al. reported printed components with single-digit micrometer lateral resolution using this novel approach [78]. 2PP involves a two-photon absorption (TPA) method to initiate photopolymerization. This nonlinear optical absorption process can greatly improve lateral and vertical resolutions at the nanoscale (100 nm or less), as photocuring mainly occurs at the laser focal spot, and any laser light out of the focal point will not initiate photocuring [79]. 2PP has been used to fabricate microdevices and sensors [80,81].

VP has been used to print ceramic components using preceramic polymers. Compared to other AM techniques, VP has the advantage of manufacturing ceramic components using preceramic material. As mentioned earlier, all AM methods follow three primary steps: creation of a digital 3D model, 3D printing, and post-processing. Additional steps may be involved depending on the type of AM process used. VP includes preparing photosensitive preceramic polymers, fabricating a green component layer by layer using an appropriate process (i.e., SLA, DLP, CLIP, or 2PP) and post-processing the green component with pyrolysis and other necessary treatments. Material preparation involves mixing the preceramic polymer with specific photoinitiators (PIs) and crosslinkers. PIs are compounds that can convert light energy into chemical energy by forming free radicals or cations when exposed to light radiation. These free radicals and cations will enable the molecular chains of preceramics to crosslink by activating vinyl groups and result in hardened and solidified structures [82]. The addition of crosslinkers to preceramic polymers can react with the molecular chains and promote the efficiency and degree of crosslinking during photopolymerization [83].

**Figure 7:**
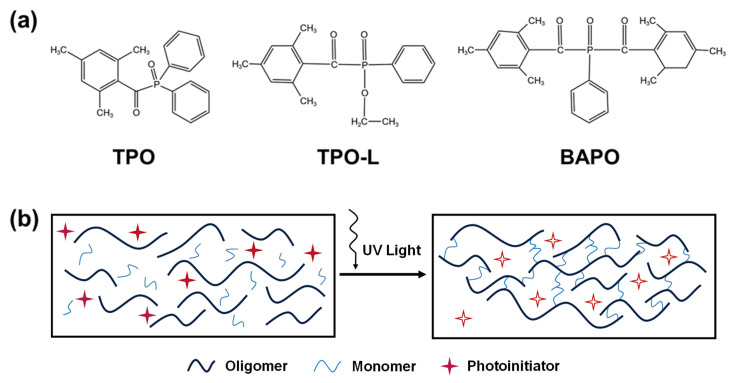
(**a**) Chemical structures of TPO, TPO-L, and BAPO; (**b**) typical photocuring process.

The choice of photoinitiators plays an essential role in vat photopolymerization where selection depends on the specific resin and printing conditions [84]. Literature search results indicate that diphenyl(2,4,6-trimethylbenzoyl)phosphine oxide (TPO), ethyl phenyl(2,4,6-trimethylbenzoyl)phosphinate(TPO-L), and phenylbis(2,4,6-trimethylbenzoyl)phosphine oxide (BAPO) are commonly used as photoinitiators for preceramic materials [85,86,87,88,89,90,91,92,93,94,95], and Figure 7a illustrates their chemical structures. TPO, TPO-L, and BAPO belong to the Norrish type I category, which can effectively generate free radicals through homolytic cleavage of their C-O or C-N bonds upon exposure to light radiation [82]. They can efficiently facilitate polymerization of different monomers and preceramic polymers to form a network structure [96,97]. Figure 7b shows a typical photocuring process.

In addition to the three main components (i.e., preceramic polymers, crosslinkers, and photoinitiators), other chemical additives are often incorporated into the photocurable resins to enhance print quality. To obtain precise geometrical structures with intricate features, free radical inhibitors and UV absorbers are typically added to absorb excess light and prevent unintended curing during vat photopolymerization [98,99]. For instance, organic dyes, such as Sudan III or Sudan Orange G, which function as UV absorbers, were used to create highly complex SiOC ceramic components [100,101].

To improve the properties of final ceramic components, reinforcements (also called fillers) can be added to resin formulations to increase mechanical, thermal, electrical, optical, or magnetic properties [102,103,104]. They can also significantly reduce shrinkage of printed parts after pyrolysis if the appropriate amount of is used [105,106]. Fillers can be inert or active depending on whether they are synthesized externally before being added to the matrix or synthesized in the matrix during the composite fabrication process [107]. Examples include particles, tubes, fibers, rods, or sheets with various dimensions [108,109].

A wide range of photocurable ceramic precursors have been explored for the formation of crosslinks. However, only a few are commercially available for vat photopolymerization. For example, Tethon 3D developed several photocurable ceramic resins for digital light processing that include Porcelite^®^, Vitrolite^®^, and alumina [110]. To expand the options available for versatile preceramics and enable practical fabrication of ceramics, extensive research has been conducted to customize the chemical compositions of photocurable formulations. Some representative preceramic materials using different vat photopolymerization processes are presented in Table 1. In the sections that follow, we discuss manufacturing ceramic components using preceramic resins using stereolithography (SLA), digital light processing (DLP), and two-photon polymerization (2PP).

(i) Stereolithography (SLA)

SLA is a vat photopolymerization process that uses a focused laser beam to selectively cure the resin and form a solid structure layer by layer. Eckel et al. prepared a UV-curable siloxane resin by mixing (mercaptopropyl)methylsiloxane, vinylmethoxysiloxane, a UV photoinitiator, a free radical inhibitor, and a UV absorber. An SLA 3D printer was used to build complex SiOC ceramic structures [98]. The results demonstrated that preceramic polymers could be used as starting materials to produce ceramics that were difficult to fabricate using conventional powder sintering techniques.

**Figure 8:**
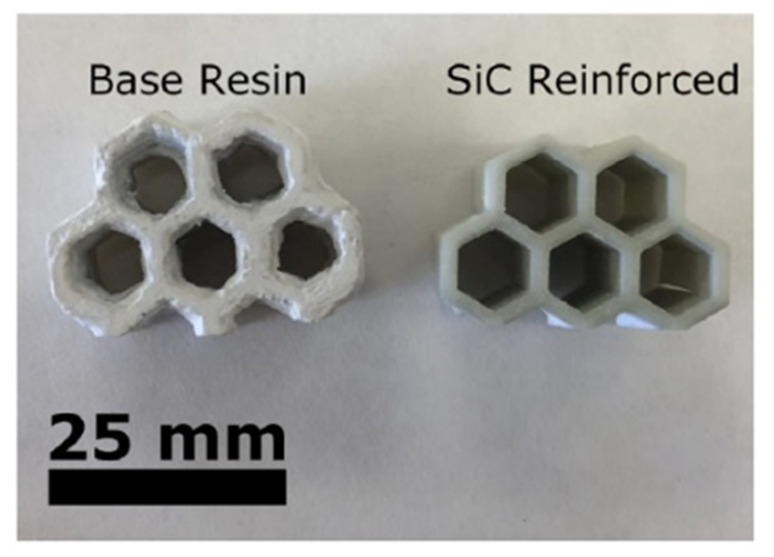
Comparison of the x–y resolution of the base resin and resin reinforced with 0.5 wt.% SiC whiskers printed using identical initial designs and printer settings. The filled resin results in a significantly less overpolymerized sample and is more defined in the x–y plane. Adapted from Reference [99] with permission. Copyright 2018 John Wiley and Sons.

Brinckmann et al. prepared a preceramic resin by mixing vinylmethoxysiloxane (VMS) preceramic polymer, co-monomer poly(ethylene glycol)-diacrylate (PEGDA), a photoinitiator, and a free radical inhibitor together [99]. To improve the quality of the final product, 0.7 wt.% of SiC whiskers (SiC_w_) was added as a filler. After printing with an SLA 3D printer, solid green components were pyrolyzed at 1000 °C into SiC whisker-reinforced amorphous SiOC ceramic composites, as shown in Figure 8. The SiC whiskers not only improved the quality of the printed part by effectively preventing over-polymerization but also decreased volumetric shrinkage of the final product by 5% compared to samples printed without SiC whiskers. Mechanical tests showed that hardness increased by 12% compared to unreinforced SiOC composite [99]. Similarly, Corcione et al. prepared a UV-curable preceramic containing multifunctional acrylic and silicone acrylate monomers [106]. Silica powder (~5 μm in diameter) was added to the resin mixture to form a UV-curable preceramic suspension. The suspension was used to print high-strength silica molds for casting aluminum using an SLA process. This research indicated that SLA 3D printing is a promising process for producing molds with complex geometries.

(ii) Digital Light Processing (DLP)

**Figure 9:**
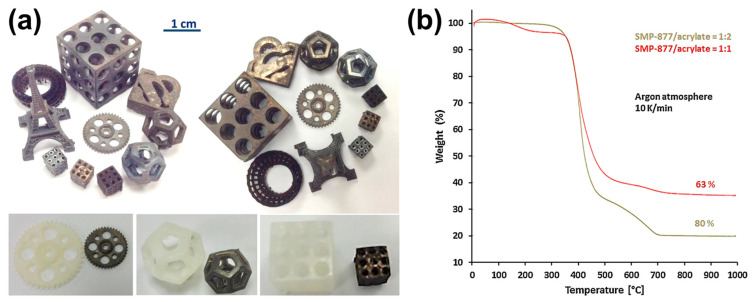
(**a**) Examples of ceramic parts derived from SMP-877/BDDA samples produced by DLP (**top**) and the comparison of printed and sintered samples (**bottom**); (**b**) thermogravimetric analysis of cured SMP-877/BDDA samples made by DLP. Adapted from Reference [83] with permission. Copyright 2017 Elsevier.

Stereolithography and digital light processing are suitable for printing ceramic components using preceramic polymers. However, DLP has some advantages over SLA, including a lower cost for 3D printers, faster printing time, and easy system maintenance. Hazan et al. fabricated SiC-rich ceramic parts using a DLP process [83]. In this research, two different commercially available polycarbosilane (PCS) preceramic liquid polymers, allylhydridopolycarbosilane (AHPCS) and allylmethylhydri-dopolycarbosilane (AMHPCS), were studied. In addition, two multifunctional (meth) acrylate monomers (1,4-butanediol diacrylate (BDDA) and 1,6-hexanedioldiacrylate (HDDA)) were also investigated as crosslinkers in the preparation of preceramic materials. Figure 9a shows the printed and sintered ceramic examples. The results showed that the quality of the 3D printed SiC-rich complex structures could be tailored by the types and ratios of crosslinkers and preceramic polymers (Figure 9b) [83]. Zanchetta et al. fabricated dense and crack-free SiOC ceramic microcomponents with cellular geometries from an engineered photosensitive polysiloxane (SILRES^®^ MK) precursor using a DLP process after pyrolysis [120].

**Figure 10:**
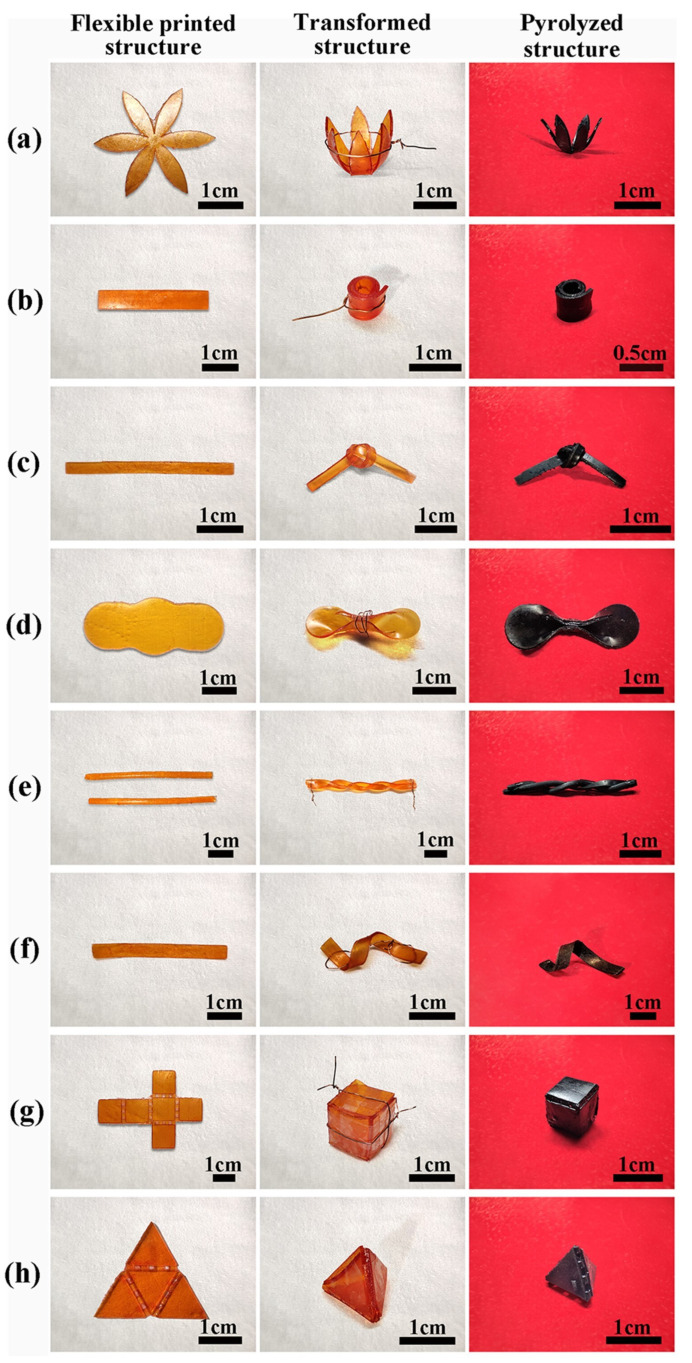
Illustration of various flexible printed structures, transformed structures, and pyrolyzed structures: (**a**) flat six-petalled flower, and the same structure transformed into a convex six-petalled flower; (**b**) flat rectangular sheet, and the same structure transformed into a cylinder; (**c**) long strip, and the same structure transformed into a knot; (**d**) thin structure, and the same structure pinched into a bow; (**e**) two long flat strips, and the same structures twisted together to form a braid; (**f**) flat rectangular sheet, and the same structure twisted into a spiral. Flat net structures and the same structures folded into a cube (**g**) and a tetrahedron (**h**). Adapted from Reference [85] with permission. Copyright 2023 Taylor & Francis.

Different reinforcements were also investigated to develop preceramics to produce high-performance ceramic components using DLP. Huang et al. formulated a preceramic mixture comprising polysilazane, trimethylolpropane triacrylate (TMPTA, a photosensitive resin monomer) and TPO (a photoinitiator) [113]. Two forms of inert Si_3_N_4_ fillers (particles and whiskers) were introduced to the resin mixtures. The resulting formulation was then loaded into a DLP printer to produce solid green components, which were subsequently converted to SiCN ceramic matrix composite after pyrolysis. A comparative study revealed that the DLP-printed ceramic matrix composite components exhibited reduced shrinkage, weight loss, and improved mechanical properties compared to their counterparts without Si_3_N_4_ fillers [113]. Precisely tailoring the percentage of reinforcement in the preceramic is critical to achieve successful prints. Excessive fillers may lead to decreased quality of the final product with increased porosity and reduced mechanical properties, primarily due to decreased continuity of the matrix phase [113]. Recently, scientists from the same research group introduced another photosensitive preceramic formulation by mixing polysilazane, vinyltrimethoxysilane, aliphatic urethane acrylate, TPO, Sudan III, and a certain amount of ɑ-Si_3_N_4_ nanopowder (0–40 wt.%) as an inert filler to make solid structures using DLP [85]. The flexible 3D printed green structures could be transformed into various shapes, and after pyrolysis, they yielded ceramic nanocomposites while retaining the initial shapes and structures, as presented in Figure 10. These results showed that the Si_3_N_4_ filler not only decreased linear shrinkage and increased ceramic yield but also enhanced the mechanical properties of the final product.

**Figure 11:**
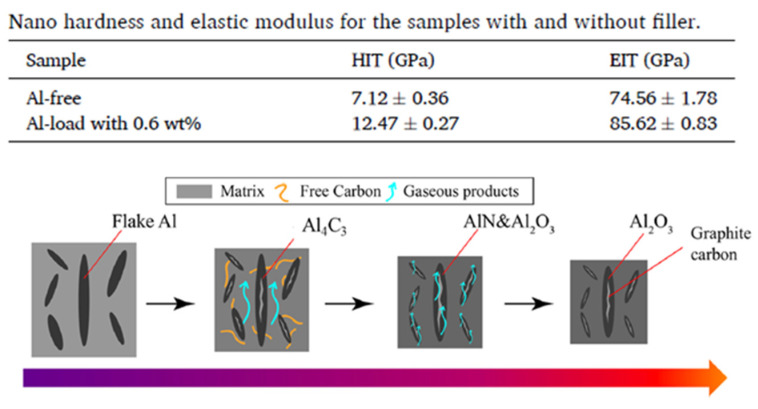
Nanohardness and elastic modulus for samples with and without filler (**upper**), and schematic diagram of the role of flaky Al filler during pyrolysis (**lower**). Adapted from Reference [121] with permission. Copyright 2023 Elsevier.

**Figure 12:**
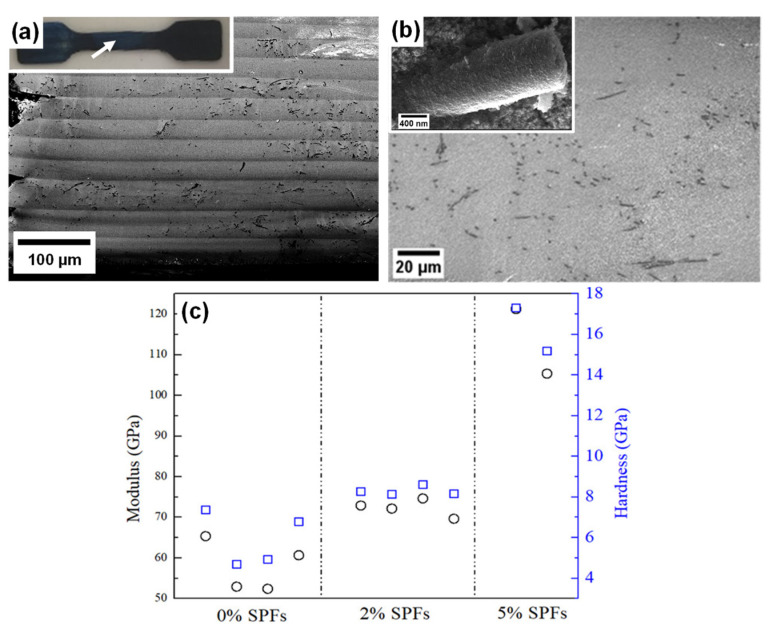
(**a**) Low-magnification image showing printed layers; (**b**) high-resolution SEM image; (**c**) tensile modulus and hardness results for SiC/C composites with a variable initial stabilized PAN fibers loading before pyrolysis. Black circle: indentation modulus, blue square: hardness. Adapted from Reference [122] with permission. Copyright 2021 John Wiley and Sons.

Introducing active fillers is another strategy to reinforce ceramic composites. With this approach, known as in-situ CMC, a chemical reaction occurs between the active filler and the matrix during pyrolysis and forms robust inert reinforcements with strong interfacial bonding with the matrix [107]. Cheng et al. discussed 3D printing of ceramics using a vinyl polysilazane-based photocurable resin mixed with varying amounts of aluminum flake (0.2–0.6 wt.%) as the active filler. After pyrolysis at 1300 °C, printed solid green parts were converted to Al_2_O_3_-reinforced CMCs. Mechanical property tests showed that printed Al_2_O_3_-reinforced CMCs (0.6 wt.% of Al flake in the preceramic precursor) had an approximate increase of 15% for elastic modulus and a ~75% increase for indentation hardness compared to the ceramics produced from Al-free precursors, as illustrated in Figure 11 (upper). This research indicated that adding a small amount of Al flake slowed crack generation. Mechanistically, Al flake gradually reacted with the oxygen contained in the TMPTA reaction monomer during pyrolysis and was later converted to hollow lamellar Al_2_O_3_ (Figure 11 (lower)). The authors believe that Al_2_O_3_ acted in two roles in the production process for high-quality ceramic components: (1) providing a channel for releasing gaseous products and (2) mechanically strengthening final products [121]. Recently, Al-Ajrash et al. developed a photocurable resin to make carbon fiber-reinforced silicon carbide composites [122]. The resin included AHPCS polymer and reactive monomer HDDA. The resin was mixed with electrospun polyacrylonitrile (PAN) nanofibers (2 or 5 wt.%) that served as carbon fiber reinforcement in the composite after pyrolysis. With the addition of active PAN fiber fillers, carbon fiber-reinforced CMCs were obtained with improved mechanical properties compared to counterparts without carbon fibers. Figure 12 illustrates the distribution of carbon fibers in the SiC composites and the mechanical test results for DLP-printed carbon fiber-reinforced CMCs [122].

(iii) Two-Photon Polymerization (2PP)

2PP is an advanced vat photopolymerization process used to produce high-resolution and precision 3D structural components for applications such as micro-electro-mechanical-system (MEMS) devices [119,123,124]. Compared to polymers commonly used for microdevices, ceramics exhibit superior mechanical properties, chemical stability, and thermal resistance [125,126]. Harnessing the distinct capabilities of 2PP printing, ceramic 3D microstructural components have been produced with preceramic materials [127]. Park et al. developed a photocurable resin, including AHPCS and organometallic (η^5^-cyclopentadienylmethyl)-trimethylplatinum (CpPtMe3), which is a multifunctional catalyst for fabricating 3D ceramic structures with near-zero shrinkage using 2PP [119]. Researchers found that adding CpPtMe3 could initiate dual crosslinking (photocuring and thermal curing), resulting in dense SiC ceramic microstructures with near-zero shrinkage after pyrolysis [119].

**Figure 13:**
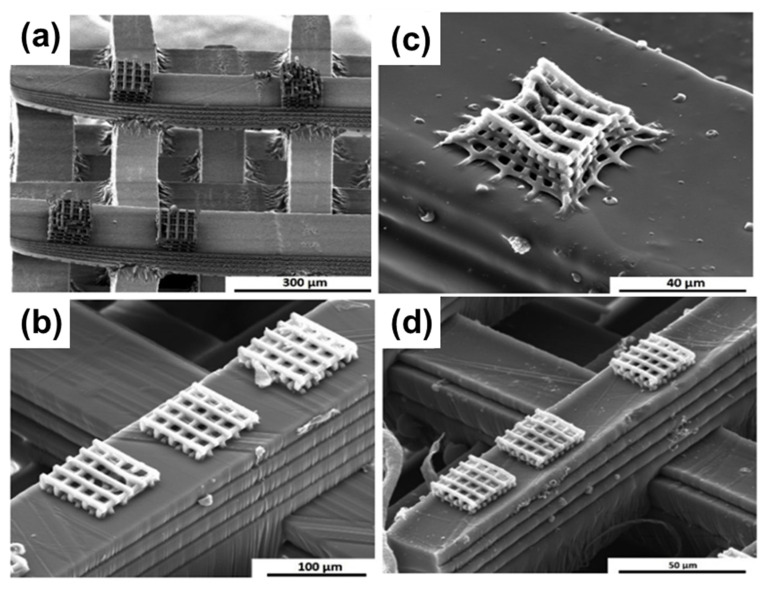
As-printed and pyrolyzed hybrid structures: (**a**) RC 711 microsized structures on SILRES^®^ MK macrosized woodpiles (as-printed); (**b**) RC 711 structures on RC 711 macrosized woodpiles (as-printed); (**c**) RC 711 microsized structures on SILRES^®^ MK macrosized woodpiles (pyrolyzed); (**d**) RC 711 structures on RC 711 macrosized woodpiles (pyrolyzed). Adapted from Reference [123] with permission. Copyright 2019 Elsevier.

Although 2PP enables high-resolution and accurate 3D printing capabilities, the process can be time consuming, especially for large-scale components [123]. To address this challenge, Schmidt et al. proposed a hybridization approach combining DLP and 2PP to facilitate rapid fabrication of multiscale dense and crack-free SiOC ceramic hybrid components using resins containing acrylate polysiloxanes [123]. With this approach, DLP was used to print 3D structures with millimeter size, and 2PP was utilized to create microstructures at a submicron size scale. Figure 13 shows as-printed and pyrolyzed solid structures containing microscopic woodpiles printed with 2PP on macroscopic rods prefabricated with DLP.

#### 3.2.2. Material Extrusion (ME)

**Figure 14:**
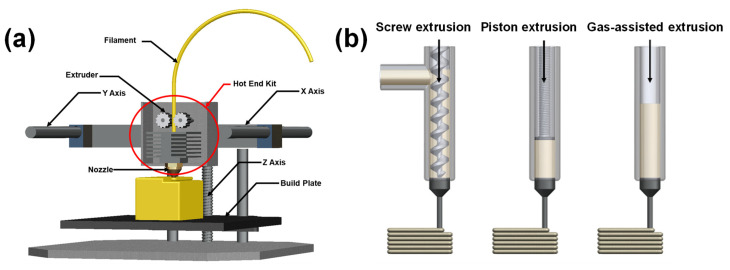
(**a**) Schematic diagram of a typical FDM 3D printer; (**b**) illustrations of three different injection systems for DIW.

Starting materials for ME can vary in form (e.g., filaments, granules/pellets, and pastes). Two common ME techniques include fused deposition modeling (FDM) and direct ink writing (DIW). FDM, also called fused filament fabrication (FFF), is the most widely adopted 3D printing method among all the available AM techniques worldwide. In this process, a flexible filament-based thermoplastic material passes through a hot nozzle and is converted to a molten state. It is then selectively deposited onto a build plate to create a solid 3D structure layer by layer. Figure 14a shows a schematic of the FDM process. In contrast, direct ink writing uses viscous pastes or granules/pellets that pass through a nozzle or orifice and are deposited on a build platform to form a 3D solid object. Depending on how feedstock material is extruded, DIW can be divided into three categories: screw extrusion-based, piston extrusion-based, and gas-assisted extrusion-based DIW systems, as shown in Figure 14b.

Material extrusion is an important 3D printing process for fabricating ceramic components using various feedstocks [128]. Using preceramic resins has particularly drawn attention because of the versatile forms of precursors, including solid filaments or pastes [129,130,131]. Solid filament containing preceramics can soften when heated and harden upon cooling, allowing for subsequent layer-by-layer deposition and solidification. Typically paste-form materials are used with DIW, where their rheological properties play a crucial role in printing high-quality components and ensure that prints maintain their solid structure and integrity during and after the fabrication process [132]. Solid-phase preceramic polymers, such as powders or those with high viscosity, may require solvents or other lower-viscosity materials to improve flow properties [102,133,134]. On the other hand, if the viscosity of the preceramic polymer is too low, particles, fibers, or other high viscous resins can be added to adjust their flow behaviors [109]. Once printed, solid green structures are converted to ceramics after pyrolysis, such as the VP process. Different feedstocks containing preceramic precursors for FDM and DIW are summarized in Table 2.

(i) Fused Deposition Modeling (FDM)

**Figure 15:**
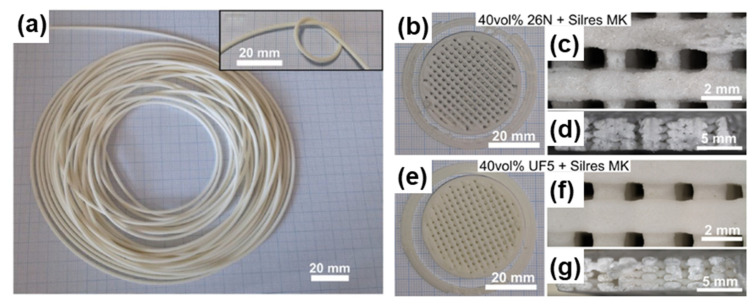
(**a**) A spool of filament made from 40 vol% UFS + SILRES^®^ MK feedstock after extrusion. The flexibility of the filament is demonstrated by twisting it in a knot without a fracture (inset); (**b**–**d**) images of porous cylinder scaffolds (before sintering) produced using filaments containing 40 vol% 26 N + SILRES^®^ MK; (**e**–**g**) 40 vol% UFS + SILRES^®^ MK. (**c**,**f**) Magnified images of the top surface of the scaffolds; (**d**,**g**) cross-sections of the structures. Adapted from Reference [129] with permission. Copyright 2019 Elsevier.

In any FDM process, the flexibility of the thermoplastic filament is essential, as it will be wound onto a spool and then fed into the extrusion nozzle. However, some preceramic polymers, especially those with high ceramic yield, tend to be brittle and are unsuitable for FDM printing. This can be possibly attributed to their high glass transition temperatures, chemical position, and molecular structures [141]. To overcome brittleness and achieve printed solid parts with structural integrity, thermoplastic preceramic polymers or mixtures containing thermoplastic polymer are used in the filaments [137]. Mei et al. developed a filament using SILRES^®^ MK to fabricate lightweight ceramic components with complex shapes [137]. Since SILRES^®^ MK resin is inherently brittle at room temperature, commercially available thermoplastic polycaprolactone (PCL) or polylactic acid (PLA) was added to the mixture to improve the filament flexibility [137]. Another study by Gojan et al. used the same ceramic precursor and formulated a thermoplastic material that can be extruded into flexible filaments for FDM [129]. Ethylene vinyl acetate (EVA) was used as an elastomeric binder to enhance filament flexibility. The authors also incorporated γ-Al_2_O_3_ particles (40 vol.%) with two different diameters (14.8 µm for 26 N and 5.3 µm for UF5) as fillers to improve the printed parts’ quality and reduce the volumetric shrinkage of the converted mullite parts after pyrolysis (Figure 15) [129].

(ii) Direct Ink Writing (DIW)

As previously discussed, the rheological properties of preceramic polymers play a critical role in the success of a DIW process [132]. Therefore, the rheological properties of the preceramic ink need to be carefully adjusted to achieve high-quality printed solid structures. Different feedstock designs have been employed for the DIW process [132]. Among them, a typical approach is to incorporate fillers to the materials to improve flowability, reduce volumetric shrinkage, and eventually enhance the properties of the pyrolyzed ceramic components [102]. For example, adding fillers to a preceramic paste usually results in a significant increase in the viscosity of the paste. However, the viscosity decreased rapidly upon the application of the shear stress, indicating a shear thinning behavior [142]. This behavior can be explained by the Herschel–Builkley model [134,143]. Non-Newtonian colloidal gels exhibit shear thinning flow behavior due to the attrition among filler particles, which can be described by the Herschel–Builkley equation: $\tau =\tau_{y}+K{\dot{\gamma }}^{n}$, where τ is the shear stress, $\tau_{y}$ is the yield shear stress, K is the visocity parameter, $\dot{\gamma }$ is the shear rate, and n is the shear thinning exponents [143,144,145]. The shear thinning effect of feedstock containing solid fillers can significantly benefit the DIW process because it allows the materials to flow smoothly through the nozzle or orifice and maintain solid structures and shapes during deposition [102,134].

**Figure 16:**
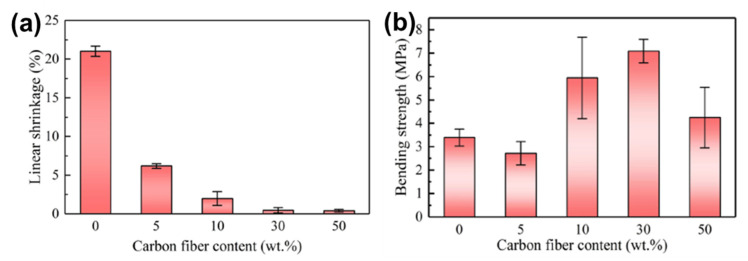
(**a**) Linear shrinkage vs. C_f_ content of the C_f_/SiC composites; (**b**) bending strength of the C_f_/SiC composites. Adapted from Reference [69] with permission. Copyright 2023 Elsevier.

Many studies have demonstrated that ceramic fillers, such as whiskers, fibers, and particles, have been mixed with preceramic material to achieve improved flowability and enhanced properties of printed ceramic products [60,68]. For instance, PCS-based suspensions containing SiC whiskers were prepared for fabricating SiC ceramic components using a DIW process [133]. The results showed that the addition of SiC whiskers not only improved the flowability of the feedstock suspension when the shear rate increased but also reduced the volumetric shrinkage of the pyrolyzed ceramic solid structures. Liu et al. prepared a mixture using PCS and chopped carbon fiber and manufactured carbon fiber-reinforced SiC composites using a DIW process [69]. The results showed that adding carbon fiber (~7 µm in diameter) helped decrease the viscosity of the materials and improve their flowability for fabricating complex solid green structures. In this study, a certain amount of n-hexane and toluene were also added as the primary and auxiliary solvents to adjust the volatility of the preceramic mixtures. After being pyrolyzed, the printed solid green structure parts were converted to carbon fiber-reinforced ceramic composites. Remarkably, the ceramic composite containing 30 wt.% of carbon fibers exhibited negligible liner shrinkage (~0.48%) and the highest bending strength (~7.09 MPa) compared to the other ceramic composites. Figure 16 shows the linear shrinkage and bending strength of the DIW-printed ceramic composites with different carbon fiber contents [69].

**Figure 17:**
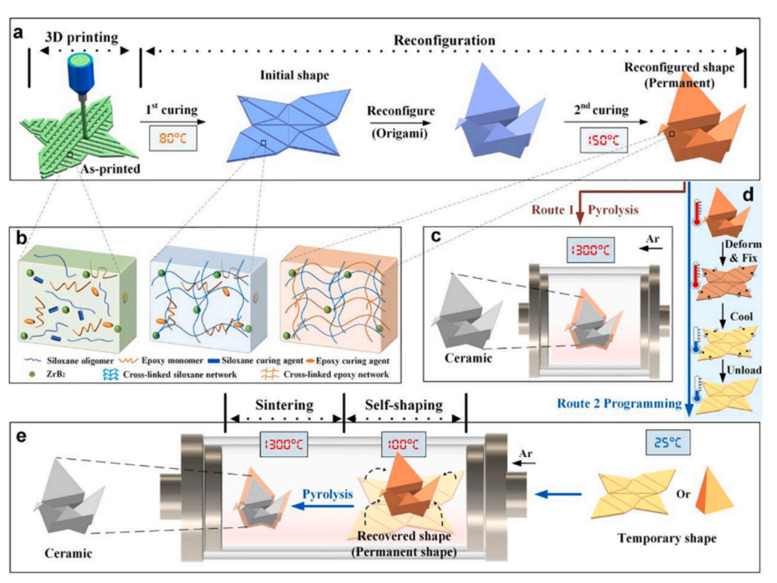
Schematic diagram of fabricating complex PDCs from reconfigurable and programmable ceramic precursors: (**a**) reconfiguration principle through two-step curing of ceramic precursors; (**b**) schematic illustration of precursor networks during the two-step curing process; (**c**) route 1 of the precursor—directly undergoing the sintering process to become ceramic; (**d**) route 2 of the precursor—folding the shape memory precursors into various temporary shapes; (**e**) the precursor within temporary shapes undergoes the recovery process as programmed and reaches the ceramic state through the sintering process. Adapted from Reference [146] with permission. Copyright 2023 Elsevier [146].

The use of micropowder or nanoparticles as fillers in the preceramic feedstock materials for DIW processes has also been studied. Chen et al. recently investigated the addition of ZrB_2_ nanoparticles (~200 nm in diameter) as an inert filler in polydimethylsiloxane for 3D printing [146]. In this study, a shape memory epoxy (SMEP) was synthesized and added to the feedstock materials to achieve reconfigurability and shape memory in the final products. The as-printed solid flat green bodies underwent a two-step curing process to make them flexible and reconfigurable. The solid structure could successively experience controllable shape transformation and then transformed to ceramic structures after pyrolysis, as shown in Figure 17 [146]. In another study, Kemp et al. developed a PCS-based mixture for the fabrication of near-net complex-shaped ultra-high temperature ceramics (UHTCs) [109]. ZrB_2_ micropowder (~1.3 µm in diameter) and SiC fiber (~10 µm in diameter and ~1 cm in length) were incorporated into the PCS polymer and resulted in the shear thinning behavior of the mixture. After pyrolysis, the as-printed solid parts were then converted to SiC composites with low linear shrinkage (<5%) [109].

**Figure 18:**
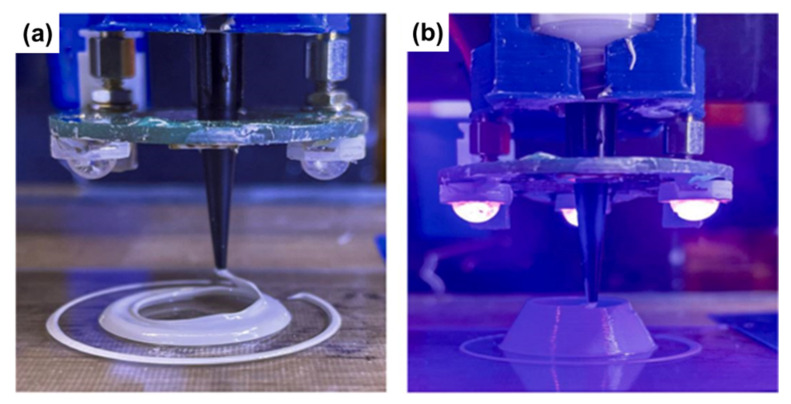
The effect of UV light on the printability of ceramic inks (**a**) without UV light and (**b**) with UV light. Adapted from Reference [147] with permission. Copyright 2022 Elsevier.

Typically, DIW-printed solid green structures using preceramic feedstocks require an additional thermal curing process to become completely solidified before they are pyrolyzed into ceramic components. This thermal curing step is necessary to produce a strong polymer green body that can withstand the stresses of subsequent processing steps [133,139]. However, alternative curing processes can also be utilized to increase crosslinking. For example, light can be used to improve solidification when appropriate PIs are applied [147]. Clarkson et al. developed a photosensitive PCS-based slurry for a UV-assisted DIW process. This slurry included AMHPCS, HDDA, and TPO-L as the preceramic polymer, crosslinker, and PI, respectively. In this research, a 50 wt.% of Si_3_N_4_ powder was selected as an inert filler, because it has good light transmission in the UV region. Figure 18 shows the UV-assisted printing process, where a 400 nm light was used for photocuring. This experiment indicated that this UV-assisted DIW process could also manufacture complex ceramic structures with low shrinkage and high conversion yield.

#### 3.2.3. Powder Bed Fusion (PBF)

**Figure 19:**
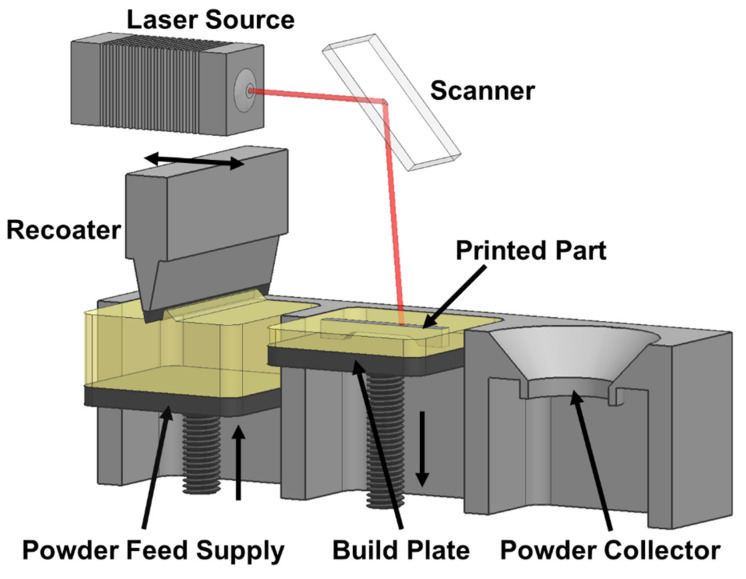
The schematic diagram of the powder bed fusion 3D printer.

PBF is an AM process that selectively fuses regions of a powder bed using thermal energy [66]. Based on how thermal energy is generated and interacts with the powder bed, PBF technologies can be categorized as laser PBF, electron beam melting (EBM), and selective heat sintering (SHS) [148]. Despite the different PBF technologies, they all operate on the same principle, i.e., thermal energy is required to fuse powders selectively on the powder bed. Laser PBF uses a laser to fuse the powder, EBM employs an electron beam, and SHS involves direct thermal heating. A typical laser PBF process and its components are shown in Figure 19. Several factors need to be considered when preceramic polymers are used for PBF processes, such as the powder size, flowability, and optical absorptivity. For instance, polymer powders usually have increased absorptivity with longer optical wavelengths [149]. Therefore, longer laser wavelengths should be considered when polymer powders are used. Friedel et al. prepared the preceramic feedstock materials by mixing SILRES^®^ MK powder (~8.5 µm in diameter) with SiC particles (~17 µm in diameter) [150]. The powder mixture was selectively cured with a CO_2_ laser (10.6 µm in wavelength), effectively forming green parts, which were subsequently converted into ceramics after pyrolysis treatment. As reported in this study, adding SiC powder to SILRES^®^ MK powder could efficiently decrease the linear shrinkage of the resulting solid parts. This research demonstrated the viability of using the laser PBF approach to print near-net-shape 3D ceramic structure [150].

#### 3.2.4. Binder Jetting

**Figure 20:**
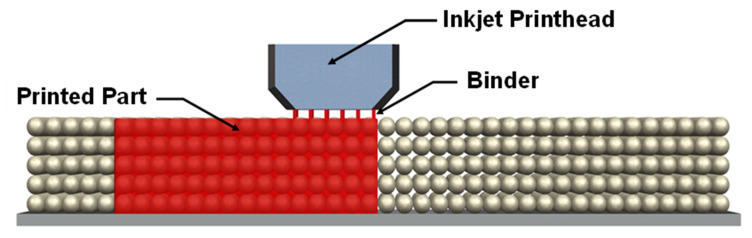
A schematic representation of binder jetting 3D printer.

BJ is an AM process that uses a liquid bonding agent that is selectively deposited to bind powder material together and form a solid part layer by layer. This process requires two materials: a powder-based material and a liquid binder material. A typical BJ process begins with creating a powder bed using a recoater. The powder bed is then selectively bonded with a binder adhesive deposited through an inkjet printhead, forming solid 3D components layer by layer, as shown in Figure 20. The BJ process can fabricate ceramic components using ceramic or preceramic powders. In one study, Zocca et al. prepared preceramic feedstocks using a commercially available PMS polymer powder for manufacturing solid parts with the BJ process [105]. To cure the PMS powder, two crosslinking catalysts (zirconium acetylacetonate (ZrAcAc) and tin-octoate (TinOc)) were employed in separate experiments. In one approach, the feedstock powder (~45–90 µm in diameter) was made from a mixed solution, including PMS polymer powder, isopropanol, and ZrAcAc. The feedstock powder was then selectively bonded by depositing isopropanol from the inkjet printhead and forming the solid green structures. In the other approach, a binder solution was prepared with TinOc and a mixture of 1-hexanol and hexylacetate. The prepared solution was then deposited on the PMS powder bed to form a 3D green structure. The fabricated green structures were further crosslinked and then pyrolyzed to obtain porous ceramic components.

## 4. Future of AM Techniques for Printing Preceramic Materials

AM is an innovative manufacturing technique that has become an integral part of Industry 4.0. Since AM process was leveraged as a manufacturing tool to fabricate complex structure prototypes, it has gradually infiltrated various industrial fields, such as aerospace, automotive, healthcare, defense, infrastructure, consumer goods, art, toy, and food, over the last decades. Many commercial market reports have shown the prevailing trends in the development of 3D printing technologies. With the development of innovative technologies such as artificial intelligence (AI), AM technologies will be advanced to a new level, which will be more intelligent, versatile, effective, functional, robust, accessible, and sustainable for fabricating high-quality, high-performance components.

AM has successfully demonstrated a promising solution in addressing the limitations of conventional manufacturing approaches and unlocking new possibilities in designing and fabricating ceramic components with desired complex and intricate structures. The unitization of preceramic polymers as feedstock materials for AM processes has shown great potential for manufacturing high-performance components. There are a few hot topics that researchers have been or will continue to work on:

(1) Advancing preceramic feedstocks for manufacturing functional components using the AM processes. With the rapid development of AM technologies and the urgent demands of advanced ceramic materials for various applications, such as high-temperature structure ceramics for hypersonic flight, electronic devices, thermal protection components, and healthcare devices, the development of preceramic feedstock materials for AM processes will continue to be one of the hot points for the fabrication of advanced ceramic components, especially the development of ceramic-based nanocomposites.
(2) Enhancing versatile and multifunctional AM processes for manufacturing high-performance functional solid components. Integrating additional features, such as thermal energy, light, ultrasound waves, or other functions, during preceramic material printing may enable high-quality products with superior performance.
(3) Developing highly dense near-net-shape advanced ceramic composites with low volumetric shrinkage and high performance is another hot topic. Volumetric shrinkage and porosity are still significant concerns for the pyrolyzed AM-fabricated ceramic components after pyrolysis, especially when preceramic polymer resins are adopted as feedstock materials. The addition of inert or active fillers or reinforcement materials has been demonstrated to effectively decrease the volumetric shrinkage of the printed ceramic components after pyrolysis and improve their properties. Exploring new composite feedstock materials for manufacturing near-net-shape high-quality ceramic components with superior performance properties using AM process is desired in the future.

## 5. Summary

AM, so-called 3D printing, is an innovative manufacturing technique for rapidly create complex structural components. It has gradually infiltrated all kinds of industrial fields over the past decades. The integration of polymer-derived ceramics with AM techniques has attracted significant attention. AM is a versatile manufacturing approach that can overcome the limits and challenges of conventional fabrication approaches for ceramics, especially for complex structures. Among all of the seven AM processes, VP and ME are the most widely used for creating ceramic components, mainly because of the nature of their feedstock material requirements. Some studies have also shown that PBF and BJ could be adopted for printing 3D solid components from preceramic resins. This article presents recent research for AM fabricated ceramics using preceramic feedstock materials. The research results demonstrated that AM processes are effective and versatile approaches for making complex structural ceramic components with extraordinary properties. The future of fabricating ceramics using preceramic feedstock materials is also discussed in this article.

## Figures and Tables

**Table 1:** Representative preceramic materials using different VP processes to make ceramics.

VP Processes	Preceramic Feedstock Material Composition	Ceramic	Ref.
SLA	Vinylmethoxysiloxane + PEGDA + SiC whisker	SiC_w_/SiOC	[99]
	Multifunctional acrylic + Silicone acrylate + SiO_2_ powder	SiO_2_	[106]
DLP	PSZ + VHPCS + PETA + HDDA + CNT	CNT/SiCN	[111]
	PVSZ + 2-Isocyanatoethyl methacrylate + SiO_2_ NP	SiO_2_/SiCN	[112]
	PSZ + TMPTA + Si_3_N_4_ powder + Si_3_N_4_ whisker	Si_3_N_4_/SiCN	[113]
	Polysiloxane (RC 711) + Al_2_O_3_ powder	Mullite	[114]
	Epoxy-acrylic siloxane (self-synthesized) + Hydroxyl silicone oil	SiOC	[115]
	Hydroxysiloxane + Al_2_O_3_ nanopowder	Mullite	[88]
	Epoxy-acrylic siloxane (self-synthesized) + ZrO_2_ micro powder + ZrO_2_ nano powder	ZrSiO_4_	[89]
	Polysiloxane + HDDA + TMPTA + Phenolic resin	SiOC	[97]
	TEOS + APTMS	SiO_2_ (transparent)	[115]
	PVSA + HDDA + TMPTA + 3-(Trimethoxysilyl) propyl methacrylate	SiOC	[86]
	Zirconium n-propoxide + Methylacrylic acid +TMPTA	ZrOC	[116]
	Polyborosilazane + Acrylate resin	SiBCN	[117]
2PP	Polysiloxane (RC 711)	SiOC	[118]
	Allylhydridopolycarbosilane	SiC	[119]

**Table 2:** Summary of the ceramics prepared based on material extrusion.

ME Processes	Preceramic Feedstock Material Composition	Ceramic	Ref.
FDM	Al_2_O_3_ powder + SILRES^®^ MK + EVA + PVA + MgO	Mullite	[135]
	Al_2_O_3_ powder + SILRES^®^ MK + MgO	Mullite	[136]
	SILRES^®^ MK + Polycaprolactone + PLA + Carbon fiber	CF/SiC + SiOC	[137]
	SILRES^®^ MK + Carbon fiber	CF/SiOC	[138]
DIW	PCS	SiC	[139]
	Calcium carbonate + Polysiloxane	β-Ca_2_SiO_4_	[9]
	Polycarbosilane + SiC whisker	SiC_w_/SiC	[133]
	PCS + SiC whisker + SiC powder	SiC_w_ + SiC_p_/SiC	[60]
	PCS + Chopped carbon fiber	CF/SiC	[69]
	PCS + ZrB_2_ + SiC fiber	ZrB_2_ + SiC_f_/SiC	[109]
	PCS + PMMA + Poly(n-butyl acrylate) + Pentaerythritol tetrakis(3-mercaptopropionate)	SiC/SiOC	[140]

## Data Availability

Not applicable.
